# Note on the Exhibition of Sulphate of Quinina

**Published:** 1847-09

**Authors:** M. Donovan


*Note on the Exhibition of Sulphate of Quinina.* By M. Donovan, Esq., M. R. I. A.—A student in medicine, M. Desvouves, some time since published, in the *Revue Médico-Chirurgicale* of Paris, a notice on an easy and certain method of removing all the bitterness of sulphate of quinina. In 1842, being at Martinique, attacked with an obstinate intermittent fever, he took sulphate of quinina which suspended the access. This medicine which, as we know, is very disagreeable to swallow, on account of its bitterness, was presented to him one day at the moment when he was going to take a cup of coffee for his breakfast.

He mixed 20 centigrammes ($3\frac{1}{2}$ grains very nearly) of sulphate of quinina in a spoonful of coffee, and swallowed it, without perceiving any bitterness. The accession of the fever being interrupted, M. Desvouves discontinued the use of the medicine; but resumed it as the disease reappeared in 1843 and 1844; and he declares that, in ten trials, the bitterness of the sulphate of quinina was entirely destroyed by the coffee, and that its febrifuge agency was in no degree impaired.

Being afterwards in attendance on a child of six years of age, M. Desvouves administered 20 centigrammes of sulphate of quinina in the same manner: but the child not being accustomed to take coffee made on water, some spoonfuls of milk were added, without any complaints of the bitterness of the medicine, although it soon triumphed over the disease. In four days, 120 centigrammes (22 grs.) were taken, and the fever did not reappear. From this it might be inferred that coffee made on water, or mixed with milk, possesses the property of removing the bitterness of sulphate of quinina, without injuring its febrifuge properties.—(*Jour. de Médecine de Mai*, 1847, *p.* 204.)

Supposing for the present that all these are well-observed facts, it might be conceived that the tannic acid present in the liquid coffee would decompose the sulphate of quinina, forming tannate of quinina, which, being an insoluble salt, would have less taste than if it were in solution; but in this respect it would have no advantage over the sulphate itself.

Decoction of coffee contains tannic acid. If sulphate of quinina be mixed with cold decoction of coffee, no change takes place; the sulphate remains undissolved. But if the decoction be very hot, the sulphate dissolves and is decomposed; the tannate of quinina partly precipitates on cooling, and partly remains suspended, forming a muddy liquid.

If to some cold decoction of coffee, a little sulphate of quinina and a few drops of dilute sulphuric acid be added, solution takes place; but that the sulphate has been decomposed will appear by the deposition of copious clouds of tannate of quinina which soon make their appearance.

Such are the facts concerned; but none of them are of any avail, either in explaining or supporting the statement of M. Desvouves. I mixed cold decoction of coffee with sulphate of quinina, and swallowed it; the taste was intensely bitter. I dissolved some sulphate of quinina in very hot decoction of coffee; the taste of this was equally bitter. I promoted the solution of the sulphate in cold decoction of coffee by means of two drops of dilute sulphuric acid; but the taste was as bitter as ever. This being my result, I forbear to theorise on the alleged destruction of the bitterness. The decoction which I used was of the usual strength made for the table. If intensely strong coffee disguise the taste of the sulphate, it must be by decomposing it, and forming the insoluble tannate.

It may be proper to observe, that there is no occasion to seek new means of disguising the bitterness of sulphate, or as it is more properly named, disulphate of quinina, at least for the use of those who can swallow a pill. For patients who cannot take it in the state of solution, which is undoubtedly the best, the form of pill is convenient. It is true there is an objection to this form, on account of the insolubility of the salt, but it is easily removed by the employment of the *neutral sulphate* of quinina, a crystallizable salt, which easily dissolves in water without any addition of acid. The neutral sulphate is in fact the preparation that should have been originally introduced into medicine, instead of the insoluble disulphate, which sometimes causes much inconvenience. The dose of both salts is much about the same; the neutral sulphate may be given with propriety in a somewhat larger quantity if the prescriber wishes to be critical.

A convenient formula would be the following: the sulphate of quinina, for the sake of clear distinction, should be marked *neutral*.

R. Sulphatis quininæ neutralis grana duodecim.
Pulveris glycyrrhizæ subtilissimi grana octodecim.
Theriacæ q. s. M. fiat massa quam divide in pilulas duodecim.

As many of these pills may be directed to be taken as the prescriber wishes: they will remain soft, and hence will have no chance of passing through the intestinal canal undissolved. No astringent substance should be contained in the pill: hence the mass must not be formed with conserve of roses. The pills may be silvered to disguise the little bitterness which otherwise they would have had.

According to M Pierquin, thirty-two grains of carbonate of magnesia conceal the taste of six grains of disulphate of quinina, without interfering with its virtues. It is also affirmed that anise or fennel perfectly masks the bitterness of this powerful febrifuge.—*Dublin Medical Press.*

